# Transcriptome-wide analysis and modelling of prognostic alternative splicing signatures in invasive breast cancer: a prospective clinical study

**DOI:** 10.1038/s41598-020-73700-1

**Published:** 2020-10-05

**Authors:** Linbang Wang, Yuanyuan Wang, Bao Su, Ping Yu, Junfeng He, Lei Meng, Qi Xiao, Jinhui Sun, Kai Zhou, Yuzhou Xue, Jinxiang Tan

**Affiliations:** 1 Department of Endocrine and Breast Surgery, The First Affliated Hospital of Chongqing Medical University, Chongqing, 400016 China; 2 Department of Orthopedic Surgery, The First Affiliated Hospital of Chongqing Medical University, Chongqing, 400016 China

**Keywords:** Cancer, Computational biology and bioinformatics, Biomarkers, Molecular medicine, Oncology, Risk factors

## Abstract

Aberrant alternative splicing (AS) has been highly involved in the tumorigenesis and progression of most cancers. The potential role of AS in invasive breast cancer (IBC) remains largely unknown. In this study, RNA sequencing of IBC samples from The Cancer Genome Atlas was acquired. AS events were screened by conducting univariate and multivariate Cox analysis and least absolute shrinkage and selection operator regression. In total, 2146 survival-related AS events were identified from 1551 parental genes, of which 93 were related to prognosis, and a prognostic marker model containing 14 AS events was constructed. We also constructed the regulatory network of splicing factors (SFs) and AS events, and identified DDX39B as the node SF gene, and verified the accuracy of the network through experiments. Next, we performed quantitative real-time reverse transcription polymerase chain reaction (qRT-PCR) in triple negative breast cancer patients with different responses to neoadjuvant chemotherapy, and found that the exon-specific expression of EPHX2, C6orf141, and HERC4 was associated with the different status of patients that received neoadjuvant chemotherapy. In conclusion, this study found that DDX39B, EPHX2 (exo7), and HERC4 (exo23) can be used as potential targets for the treatment of breast cancer, which provides a new idea for the treatment of breast cancer.

## Introduction

Invasive Breast cancer (IBC) has the highest morbidity rate and second highest mortality rate after lung cancer in women in the USA^1^. The 5-year survival of IBC was 85% from 2010 to 2014^2^. The mortality rates have recently improved, while the median survival in the metastatic setting is still dramatically low (median, 24 months)^3^.

Molecular classification of IBC has been widely applied in the clinical setting^4,5^. It has been providing great guidance for individualized treatment, including surgery, endocrine therapy, chemotherapy, and various types of targeted therapy, which significantly improves disease-free survival in patients^5^, but this classification is still relatively difficult to use for describing tumour progression in individuals^6,7^. Nowadays, gene expression profiles have been developed to serve as a complement for molecular classification and gain additional prognostic assessment for precise therapy; for example, the Oncotype DX Recurrence Score has been applied to determine the individual recurrence risk in hormone-receptor-positive patients^8^. However, the change in molecular subtyping still tends to be highly unpredictable for tumour heterogeneity as the long-term effect of dynamic epigenetic regulation occurs in tumour cells in the treatment process^9,10^, which make it highly difficult for prognosis assessment and selection of proper therapeutic schedule in clinical application^10^.

Alternative splicing (AS) is a crucial process of forming mature mRNAs from pre-mRNAs, which induces generation for variation of transcript and diversity of proteome^11^. AS events are usually regulated by splicing factors (SFs), which function by influencing transcription start site choice and making decisions of gaining and losing exons^12^. AS events are currently categorized into seven transition types: alternative acceptor site (AA); alternative donor site (AD); alternative promoter (AP); alternative terminator (AT); exon skip (ES); retained intron (RI); and mutually exclusive exons (ME)^13–15^. In tumour cells, both somatic mutations and dysregulation of SFs could activate the AS process^16–18^. AS and SFs have been proved to be important components of tumour initiation, progression, metastasis, and therapeutic resistance^19–22^. They induce splicing isoforms that switch under different stresses and environments and then form a set of affect domains in proteins, leading to functional transform for tumour adaptation. AS prognosis modelling of several cancers have been proposed; however, all of them are still in the research stage and need further experimental evidence for verification^23–26^.

In this study, we modelled the survival-related AS prediction scores in IBC using genome-wide AS events and corresponding clinical information and conducted corresponding experimental verification. Moreover, we revealed the potential AS events for evaluating the efficacy of neoadjuvant chemotherapy and SFs for regulating the expression of AS events. Our results provide a new model that involves 14 AS events that serve as potential targets for individual precise treatment in IBC.

## Methods

### Design and process of the study

**Figure 1:**
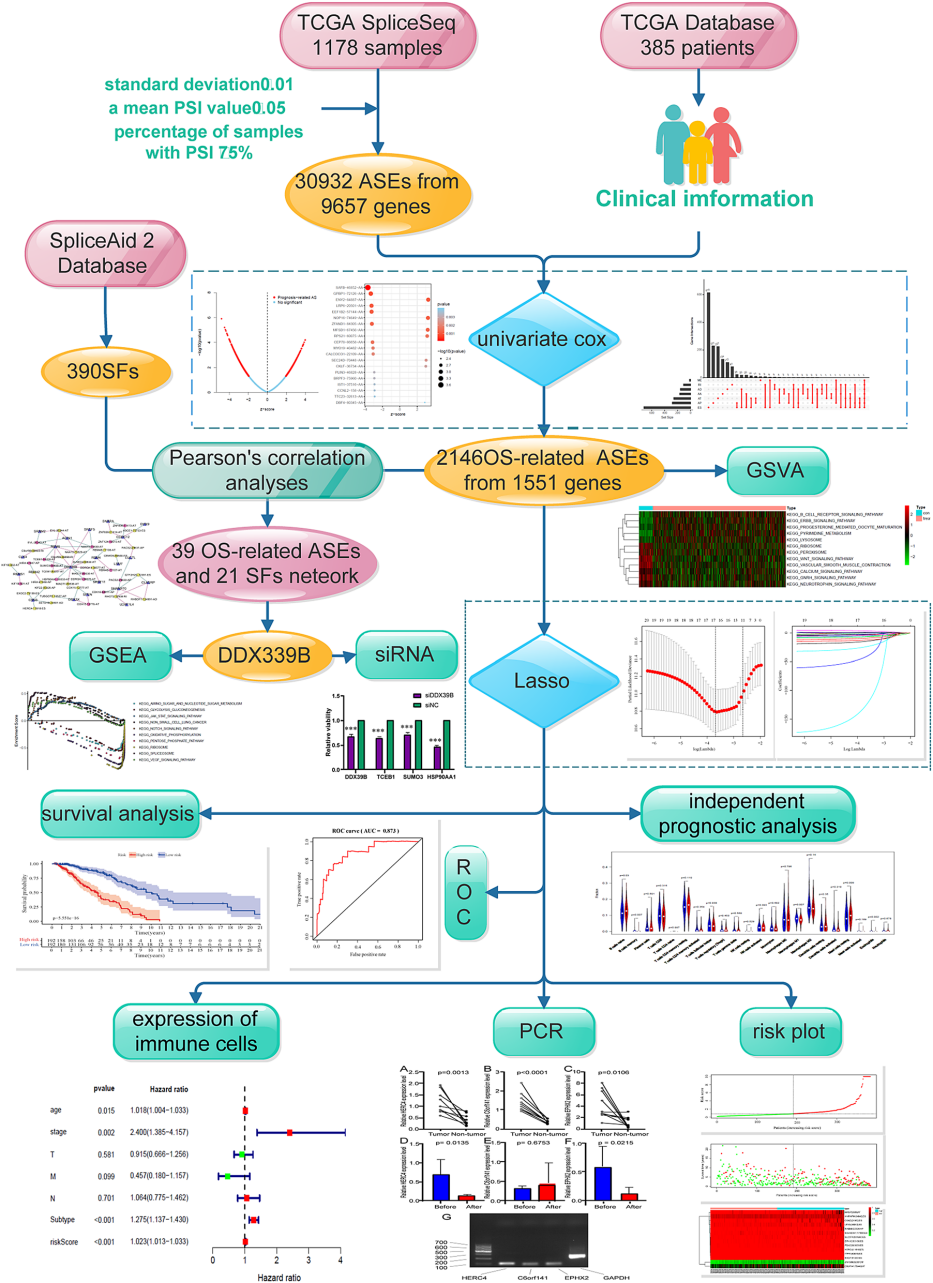
Overall flowchart of the AS event prognostic model construction and verification.

Shown as a schematic flowchart (Fig. 1). AS events and clinical information of patients were collected from The Cancer Genome Atlas (TCGA) database and strictly filtered. Then, a series of bioinformatics methods were applied, and prognostic features of AS were constructed for patients with IBC. The expression of three AS events and their parental genes were evaluated by applying quantitative real-time reverse transcription polymerase chain reaction (qRT-PCR). Finally, the correlation between AS occurrence and SFs were obtained by Pearson correlation analysis. Knockdown of node SF by small interfering RNA (siRNA) and we observed the changes of three survival-related shear events interacting with the node SF.

### Data download and preparing

RNA sequencing expression data of the IBC dataset was downloaded from TCGA (https://portal.gdc.cancer.gov/ accessed July 27, 2019). TCGA is a publicly available database that contains genomic, epigenomic, and transcriptomic information of > 33 cancer types. Samples were acquired, and each sample was matched with its corresponding genes and expressions. AS data of IBC samples are presented as percent spliced in (PSI) values. They were obtained from TCGA SpliceSeq, which is a database for searching alteration of mRNA splicing patterns in tumour and normal samples^27^ (https://bioinformatics.mdanderson.org/TCGASpliceSeq/). Samples with > 30% of missing PSI values were excluded. Clinical information on the IBC dataset was also downloaded from TCGA (accessed August 1, 2019). Data matching and filtering were performed using Bioconductor R packages (version 3.6.1). The inclusion criteria were as follows: standard deviation > 0.01, mean PSI value > 0.05 of AS events, and percentage of samples with PSI value > 75%. Data imputation was conducted by applying the k-nearest neighbour method.

### Survival-related AS events identification and gene set variation analysis (GSVA)

Overall survival-related AS events were screened by performing a univariate Cox regression analysis using the Survival R packages (3.6.2) (*P* ≤ 0.05). The interactive sets between AS events of seven subtypes were displayed in an UpSet plot drawn using Upset R package (version 1.3.2). The significance of survival -related AS events in each AS type was ranked and shown in bubble plots using Survival R package.

GSVA is a non-parametric and unsupervised method to estimate the function of gene set from sample expression data. The ‘GSVA’ package^28^ in R language was used to evaluate the different functions of parent genes of survival related AS events between high and low risk groups. The screening criteria for GO and KEGG were |logFC|> 0.45, adjusted *P* < 0.05, and |logFC|> 0.2, adjusted *P* < 0.05, respectively. And the enrichment results were visualised using the ‘pheatmap’ package (https://CRAN.R-project.org/package=pheatmap).

### Prognostic model construction and independent prognostic analysis

The least absolute shrinkage and selection operator (LASSO) method was applied to construct the prediction model. The LASSO method selects the most powerful prognostic predictors by forcing absolute value of the regression coefficients to be less than the constant value. It could effectively avoid overfitting of the model and filter top significant AS events. The LASSO method was applied using the glmnet package^29^.

The prognostic model was presented as a risk score, which is estimated by the sum of PSI value of each AS event multiplied with its corresponding coefficient (α). It was thus obtained using the following formula: risk score = PSI of AS1 × α1AS1 + PSI of AS2 × α2AS2 + …PSI of ASn × αnASn. Then, the patients were divided into high- and low-risk groups according to their median risk score for effectiveness validation of the model. The Kaplan–Meier analysis was applied to show the survival difference of the low- and high-risk groups. Then, the distribution of risk stratification and time-dependent receiver operating characteristic (ROC) analysis was performed as the area under the curve (AUC) to evaluate the quality of model using timeROC R package (version 0.3)^30^. Risk curve and scatter plot also showed the risk score and vital status of individuals. The heatmap revealed the dynamic change in the PSI in top prognosis-related AS events as the risk increases.

In order to observe the influence of age, TNM stage, tumour classification/type and risk score on the prognosis of patients, we used R language to draw univariate and multivariate forest maps containing these factors.

### Analysis of the difference of immune cells between high and low risk groups

Based on transcriptome data, CIBERSORT deconvolution algorithm was used to quantify the relative proportion of 22 kinds of immune cell, such as naive B cells, memory B cells, plasma cells, CD8 + T cells, naive CD4 + T cells, resting memory CD4 + T cells, activated memory CD4 + T cells, T follicular helper cells, regulatory T cells (Tregs), gamma delta T cells, resting natural killer (NK) cells, activated NK cells, monocytes, M0 macrophages, M1 macrophages, M2 macrophages, resting dendritic cells, activated dendritic cells, resting mast cells, activated mast cells, eosinophils, and neutrophils. Wilcoxon test was used to compare the difference of immune cells between high- and low-risk group.

### Construction of the splicing correlation network

The data of SFs were downloaded from the SpliceAid 2 database^31^, and the expression of IBC survival-related AS events was accessed from RNA-sequencing data. Pearson’s correlation analyses were employed to select the SF genes that were significantly associated with the PSI of survival-associated AS events. The inclusion criterion was that the correlation coefficient was ≥ 0.6 or ≤  − 0.6 (*P* < 0.001). The regulatory network between SFs and AS events is generated using Cytoscape (version 3.4.0).

### Gene set enrichment analysis (GSEA) and molecular docking

To further study the potential biological function of node SF in the occurrence and development of breast cancer, we divided all breast cancer samples into two groups according to the expression of node SF. Then, GSEA software (version 4.0.3) was used to perform Gene ontology (GO) and Kyoto Encyclopedia of Genes and Genomes (KEGG) enrichment analyses under the condition *P* < 0.05.

The secondary and tertiary structures of AS events exons related to node SF were formed by using the online program RNAfold (https://rna.tbi.univie.ac.at/cgi-bin/RNAWebSuite/RNAfold.cgi) and RNAComposer (https://rnacomposer.ibch.poznan.pl/). The crystal structure of the node SF was obtained from Protein Data Bank (https://www.rcsb.org/), and the online tool HDock (https://hdock.phys.hust.edu.cn/) was used to predict and visualize the nucleic acid-protein structures.

### Cell culture

MDA-MB231 (Claudin-low, ER-, PR-, HER2-) human breast cancer cells lines were obtained from the ATCC. Cells were maintained in the complete DMEM medium (high glucose Dulbecco’s modified Eagle’s medium) (Gibco), supplemented with 10% heat inactivated foetal bovine serum (FBS) (Gibco) and 1%penicillin/streptomycin (Gibco) and the cells were incubated at 37 °C, 5% CO2 and 70% humidity.

### Patients

We collected 10 IBC samples and corresponding para-cancerous samples from patients who underwent modified radical mastectomy for IBC after preoperative treatments of neoadjuvant chemotherapy scheme (docetaxel combined with epirubicin and cyclophosphamide, TEC) of four times and were evaluated according to Response Evaluation Criteria in Solid Tumours (version 1.1 criteria) from May 2019 to January 2020 in the department of Endocrine and Breast Surgery of the First Affiliated Hospital of Chongqing Medical University. Tissues were excised and transferred in liquid nitrogen for further assessments. All patients were informed and provided written informed consent. All patients were diagnosed with molecular classification of triple-negative BC and had no evidence of distant metastasis, certified by two pathologists in our department. And only patients with TNM stages I–II were included in the study. Patients were divided into partial response (PR) and stable disease (SD) groups according to their response to chemotherapy. The response of patients to NACT was selected according to the sum of the maximum diameters of target lesions measured by type-B ultrasonic in New Guidelines to Evaluate the Response to Treatment in Solid Tumors. In SD group, the change of the sum of the maximum diametral of target lesions was between the reduction of ≥ 30% and the increase of ≥ 20%. In PR group, the sum of maximum diameters of target lesions decreased ≥ 30%. Requisite original clinical data were acquired from pathology reports and hospital records.

### RNA interference

Cells are seeded in proper density and incubated for 24 h, then siRNA of DDX28B (synthesized by Ribobio, concentration is 25 nM) (Guangzhou, China) were transfected with OPTI-MEM (31985070, Thermo Fisher) Lipofectamine and RNAiMAX (13778–150, Thermo Fisher, Rockford, IL, USA) by instruction, supernatants were removed and new medium were changed 24 h later. Cells were harvest 72 h later for further experiments. Target sequence for siRNA: GCAGCAGTACTACGTGAAA.

### Total RNA extraction

Total RNA from IBC tissues and cultured cells was extracted using an UNIQ-10 column Total RNA Extraction Kit (Sangon Biotech). The RNA concentration and purity were assessed using a SMA4000 microspectrophotometer (Merinton Instrument, Inc.) and applying RNA electrophoresis by DYY-6C electrophoresis apparatus (Liuyi, Beijing).

### Reverse transcription and qRT-PCR quantification

RNA from human IBC tumour tissues were reversed-transcribed using a RR047A cDNA synthesis kit (TaKaRa, China). qRT-PCR was performed for C6orf141 (exon3; forward primer, TTGGCCTGGTTGAAGCTCTG; reverse primer, TCAGGTGGTAAAGTCAAGCAGT; product length, 71), HERC4 (exon23; forward primer, GGACCTCCATTTTCCTTTGGC; reverse primer, TCCC AACATCAGGCATAGTCT; product length, 92), EPHX2 (exon 7; forward primer, GTCCGTCTGCATTTTGTGGAG; reverse primer; CCAACTCTCGGGAA ATCCAT; product length, 72), C6orf141 (forward primer, AGGAGCCCAACTACCCTTCT; reverse primer, TCCTCAGTCCTCGTGGTCAT), HERC4 (forward primer, CCTATAATGGGCAGTGTCTACCA; reverse primer, CACAGTTCTGGGGACTAGAGT), EPHX2 (forward primer, TGACAGTGAAGCCAGGGATC; reverse primer, ACCATCTCCTCACACAGCAA), ELOC (forward primer, CATCAGGCACGATAAAAGCCA; reverse primer, GCTGTTAGTGTAGCGAACCTTG), DDX39B (forward primer, TCCAGGCCGTATCCTAGCC; reverse primer, GCATGTCGAGCTGTTCAAGC), HSP90AA1 (forward primer, CATAACGATGATGAGCAGTACGC; reverse primer, GACCCATAGGTTCACCTGTGT), and SUMO3 (forward primer, GACACCATCGACGTGTTCCA; reverse primer, CGGGCCCTCTAGAAACTGTG) using a 2X SG Fast qPCR Master Mix (High Rox, B639273, BBI) with the StepOnePlus fluorescence quantitative PCR instrument (ABI, Foster, CA, USA). GAPDH (forward primer, GCCCGTTTGCATTTTGTGGAG; reverse primer, CCAACTTTCGGGAA ATCCAT; product length, 126) was used as a gene for internal control, the products of exon-specific qRT-PCR were conducted with electrophoresis with condition of 1.5% agarose, 1*TAE, 150 V, 100 mA, and 20 min, T-tests and paired t-test was conducted using Graphpad Prism 8.0.

The study protocol and all methods which were performed were approved by the ethics committee of Affiliated Hospital of Chongqing Medical University (2020–155), all methods were confirmed to be performed in accordance with the relevant guidelines and regulations.

### Ethical approval and consent to participate

This study was approved by the Ethics Committee of the First Affiliated Hospital of Chongqing Medical University, and each participating patient provided written informed consent.

## Results

### Overview and function of AS events profiles in TCGA IBC cohort

**Figure 2:**
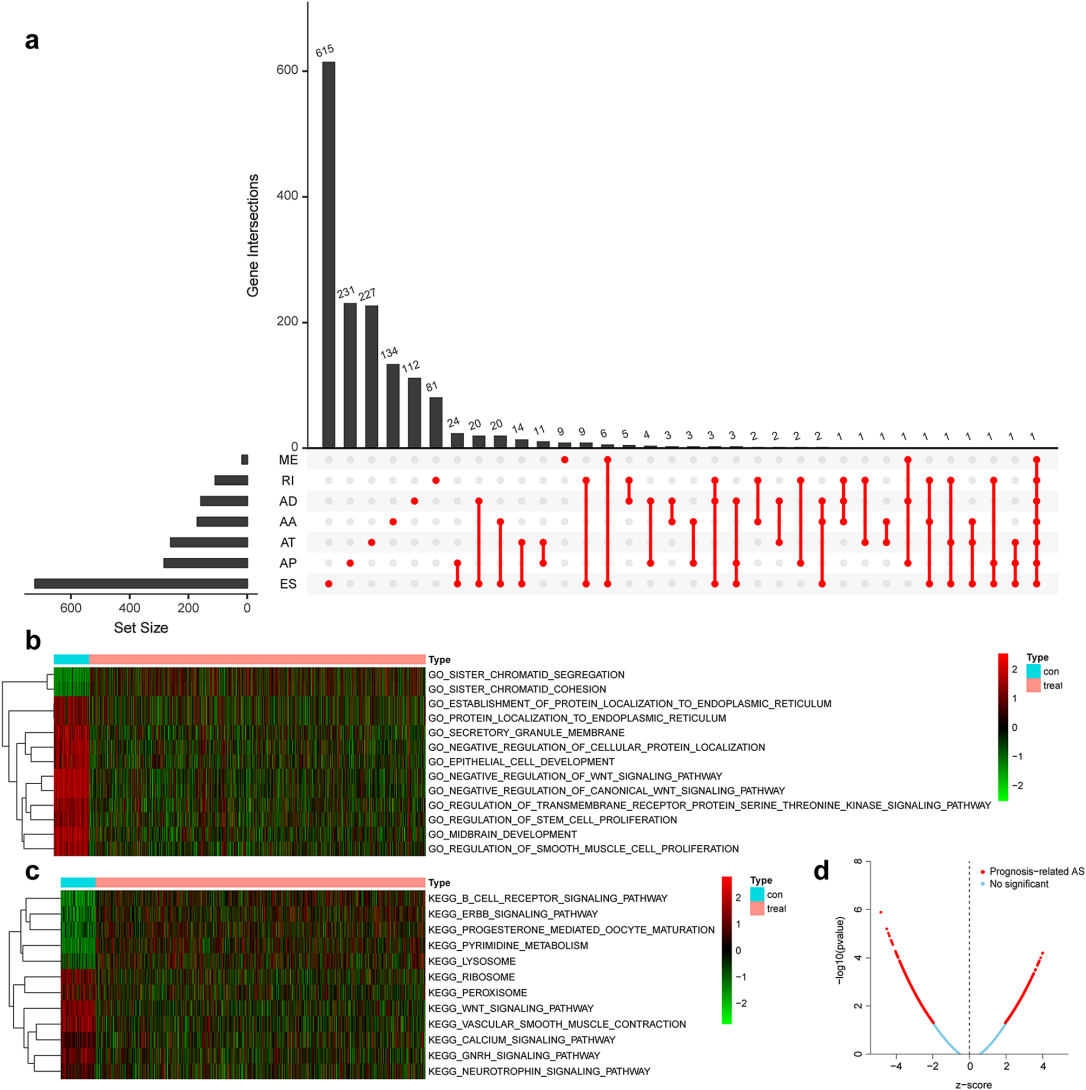
Distribution and function of survival related AS events. (**a**) Overview of AS events profiling in the IBC cohort. The red dots indicate the intersection between gene set and different AS event type. *AA* alternate acceptor; *AD* alternate donor; *AP* alternate promoter; *AT* alternate terminator; *ES* exon skip; *ME* mutually exclusive exons; *RI* retained intron. (**b**) Gene ontology results of gene set variation analysis. (**c**) Kyoto Encyclopedia of genes and genomes results of gene set variation analysis. (**d**) Volcano plot of the survival-associated and insignificant AS events. Red dots represent the survival-related AS events and the blue dots represent the AS events that has no significant relation with prognose of patients.

**Figure 3:**
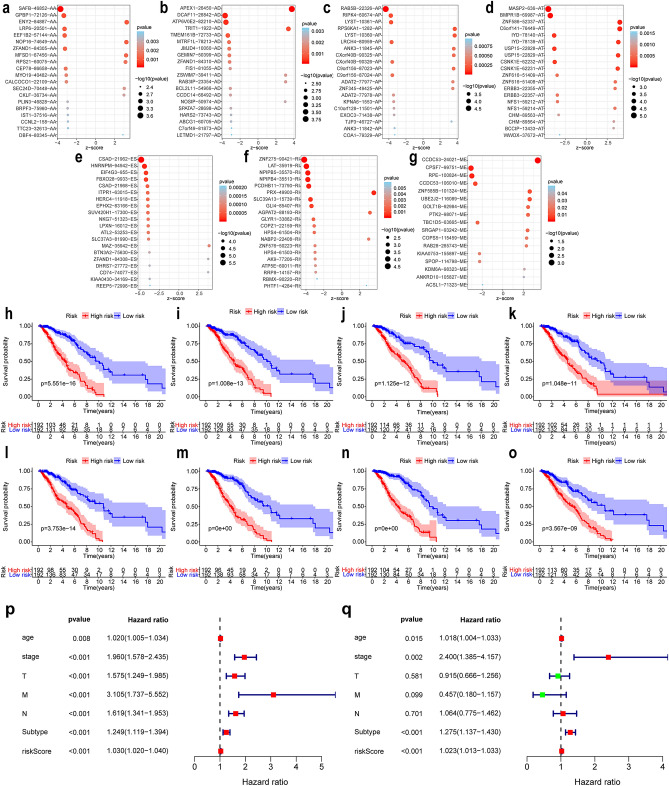
(**a**–**g**) Bubble plots of the top 20 most significant survival-related AS events in seven AS subtypes. AS subtypes of AA, AD, AP, AT, ES, RI, and ME, respectively. AS events are considered protective when Z < 0, and the opposite when Z > 0. The size and colour of the bubble show the significance of each AS event. (**h**–**o**) Kaplan–Meier survival curve of prognostic related AS events in IBC. **h**–**o** represents the survival curve of total AS and AA, AD, AP, AT, ES, RI and ME subtypes events, respectively. (**p**–**q**) independent prognosis analysis. (**p**) univariate independent prognosis analysis. (**q**) multivariate independent prognosis analysis. The red square represents HR > 1, and the green square represents HR < 1. AA: alternate acceptor; *AD* alternate donor; *AP* alternate promoter; *AT* alternate terminator; *ES* exon skip; *ME* mutually exclusive exons; *RI*: retained intron.

AS events profiles of 385 patients with IBC, including 249 (64.68%) alive and 136(35.32%) dead patients, from the TCGA database were extensively analysed. The median follow-up period was 1378.2 days, ranging from 92 to 8088 days. According to the articles by Thorsson V et al^32^, we obtained the tumour classification information of these samples, including 172 cases of lumA, 59 cases of lumB, 26 cases of Her2, 61 cases of basal, 64 cases of normal, and 3 cases of unknown typing; and according to the articles of Cancer Genome Atlas Network^33^, we obtained 217 cases of positive ER status, 66 cases of negative status, 192 cases of positive PR status, 92 cases of negative status, 41 cases of positive Her2 and 233 cases of negative typing in these samples. In clinical characteristics, TNM staging was complete in 321 cases. A total of 30,932 AS events with 9657 parent genes were detected; 16,381 AS events with 7635 genes remained after filtering; a single gene is estimated to have 2.15 types of AS events on average. A total of 2146 survival-related AS events from 1551 genes were identified by Cox univariate analysis. In detail, we assessed 863 ESs in 721 genes, 167 ADs in 156 genes, 171 AAs in 168 genes, 118 RIs in 107 genes, 395 ATs in 259 genes, 415 APs in 281 genes, and 17 MEs in 16 genes that are related to survival. The UpSet plot (Fig. 2a) demonstrated that one gene might contain all AS types, and ES was the most prevalent type of AS, while ME was the least. The volcano plot suggested that a majority of AS events were associated with survival in IBC (Fig. 2d). The top 20 most survival-associated AS events in seven subtypes were respectively presented in bubble plots (Fig. 3a–g).

Then 1551 parental genes of 2146 survival-related AS events were analysed by GSVA enrichment analysis to explore the biological characteristics of these genes in cancer and paracancerous tissues. Figure 2b,c showed that B cell receptor signaling pathway, ERBB signaling pathway, progesterone mediated oocyte maturation, pyrimidine metabolism, and lysosome were highly expressed in cancer tissues, which had effect on promoting cancer. However, ribosome, Peroxisome, Wnt signaling pathway, vascular smooth muscle contraction, calcium signaling pathway, Gnrh signaling pathway and neurotrophin signaling pathway were low expressed in cancer tissues, showing inhibitory effects on cancer.

### Construction and verification of survival related AS event prognostic model

**Figure 4:**
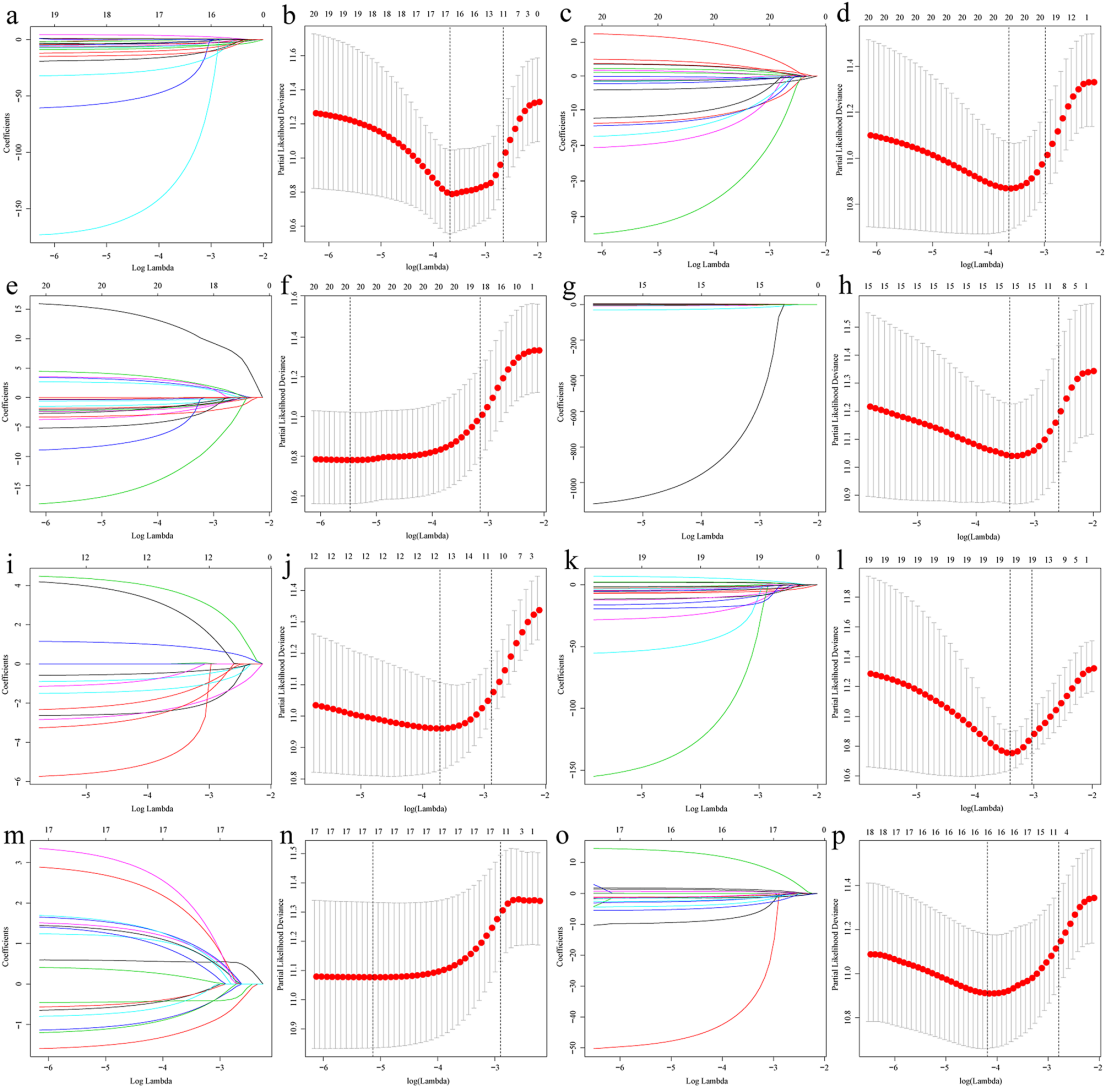
Multivariate prognostic model constructed by LASSO regression. (**a**, **c**, **e**, **g**, **i**, **k**, **m**, **o**) show the coefficients in the LASSO regression for survival-related screening of AS in general and the subtypes of AA, AD, AP, AT, ES, RI, and ME, respectively. (**b**, **d**, **f**, **h**, **j**, **l**, **n**, **p**) show the cross-validation for tuning parameter selection in the proportional hazards model in the AS in general and subtypes of AA, AD, AP, AT, ES, RI, and ME, respectively. *LASSO* least absolute shrinkage and selection operator; *AA* alternate acceptor; *AD* alternate donor; *AP* alternate promoter; *AT* alternate terminator; *ES* exon skip; *ME* mutually exclusive exons; *RI* retained intron.

The whole and seven subtypes of AS events were analysed by LASSO, respectively (Fig. 4). 93 prognosis-related AS events were identified in subtypes (Supplementary Table S1), and the overall AS type prognostic model contained 14 top significant prognosis-related AS events, including CSAD|21962|ES, MASP2|636|AT, HNRNPM|94942|ES, RAB5B|22326|AP, FBXO28|9933|ES, ITPR1|63015|ES, HERC4|11918|ES, ZNF586|52337|AT, C6orf141|76449|AT, EPHX2|83166|ES, SUV420H1|17300|ES, NKG7|51323|ES, LPXN|16012|ES, and SLC37A3|81990|ES (Table 1). The risk score of each patient is calculated based on the PSI values of these AS events. Then, patients with risk scores higher than the median of 0.7555 were allocated to the high-risk group, whereas the remaining patients were assigned to the low-risk group.

**Table 1:** Multivariate prognostic model containing 14 survival-associated AS events.

Id	Coef	HR	*P* value
CSAD|21962|ES	− 4.174	0.015389	4.60E−05
MASP2|636|AT	− 4.564	0.010416	2.87E−05
HNRNPM|94942|ES	− 5.956	0.00259	0.052207
RAB5B|22326|AP	− 5.513	0.004034	0.029795
FBXO28|9933|ES	− 18.986	5.68E−09	0.005594
ITPR1|63015|ES	− 56.278	3.62E−25	4.06E−06
HERC4|11918|ES	− 32.543	7.36E−15	1.49E−06
ZNF586|52337|AT	4.934	138.9373	0.003321
C6orf141|76449|AT	0.981	2.666126	0.034518
EPHX2|83166|ES	− 16.046	1.08E−07	0.001239
SUV420H1|17300|ES	− 9.282	9.31E−05	0.003428
NKG7|51323|ES	− 168.188	9.06E−74	0.001175
LPXN|16012|ES	− 6.593	0.001369	7.76E−05
SLC37A3|81990|ES	− 11.881	6.92E−06	0.001907

**Figure 5:**
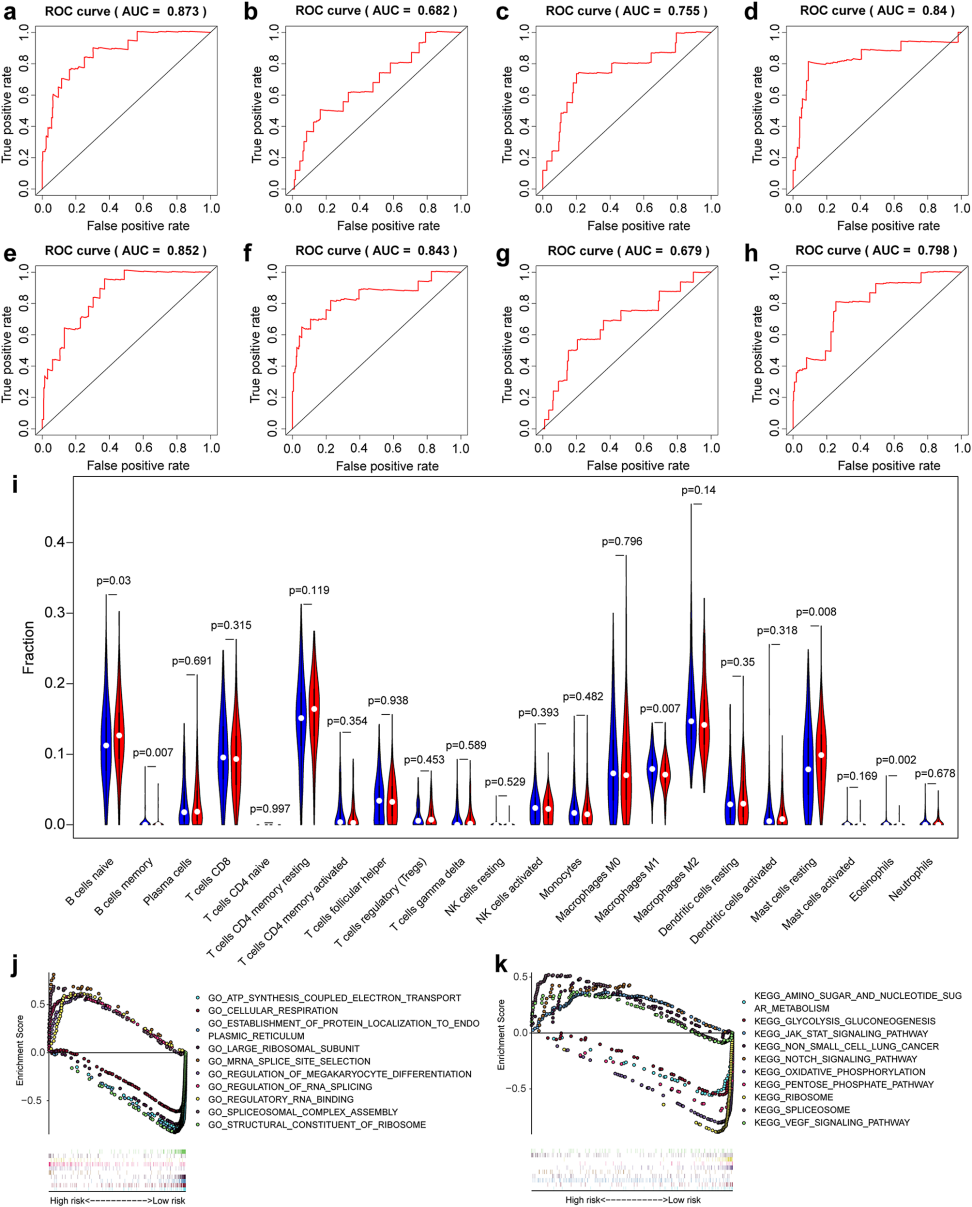
(**a**–**h**) ROC curves of prognosis risk score. (**a**–**h**) Represents the ROC curves of total AS and AA, AD, AP, AT, ES, RI and ME subtypes events, respectively. (**i**) The relationship between different risk groups and immune cell infiltration. (**j**, **k**) Gene set enrichment analysis of DDX39B. *AA* alternate acceptor; *AD* alternate donor; *AP* alternate promoter; *AT* alternate terminator; *ES* exon skip; *ME* mutually exclusive exons; *RI*: retained intron.

**Figure 6:**
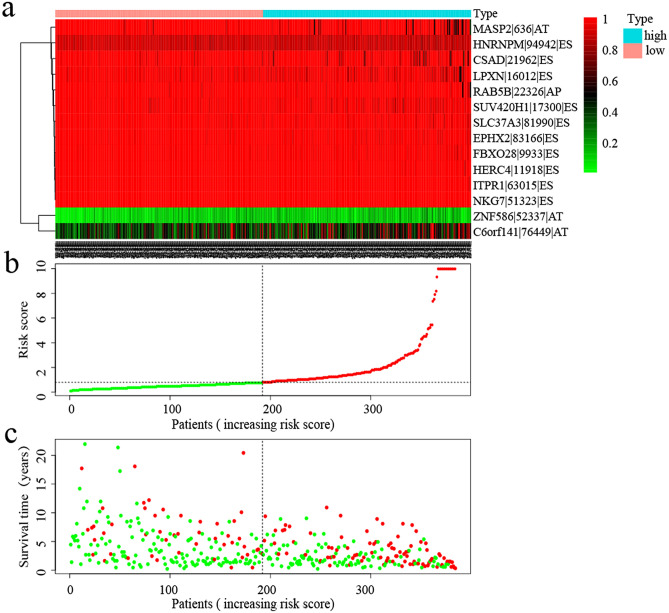
Analysis of risk score prognostic model based on the survival-associated splicing events. (**a**–**c**) Respectively represents the expression of prognostic-related AS events, risk score, and patients’ survival status between the high and low risk groups. In the risk curve (**b**), the green dots indicate the patients in low risk group and the red dots indicate the patients in high risk group. In the scatter plot (**c**), the green dots represent living patients and the red dots represent dead patients.

Kaplan–Meier (K-M) analysis was performed to verify the relationship between the signatures and survival of patients. As shown in Fig. 3h–o, patients with high risk scores had relative low survival probabilities compared to patients with low risk scores. Then, we used ROC curve analysis to assess the prognostic power of the final model. The AUCs of the ROC curves were all > 0.6 (Fig. 5a–h), revealing their powerful prognostic abilities. Furthermore, risk curve and scatter plot showed the risk score and vital status of each patient, and the heatmap showed that ZNF586|52337|AT and C6orf141|76449|AT are risk events and NKG7|51323|ES is the most protective event (Table 1 and Fig. 5). It also seemed that patients with higher risk scores likely expressed a lower PSI value of the protective AS events in general (HR < 1) but a higher PSI value of risky AS events (HR > 1) (Fig. 6).

### Screening of independent risk factors and characteristics of immune infiltration among different risk groups

As shown in Fig. 3p,q, the *P* values of age, stage, subtype, and risk score in the univariate and multivariate independent prognostic analysis were all less than 0.05, indicating that these factors could be used as independent factors.

The results of immune cell set expression among different risk groups showed that, naive B cells, resting mast cells was highly expressed in the high-risk group and, memory B cells, M1 macrophages and eosinophils was highly expressed in the low-risk group (Fig. 5i).

### Validation of exon and gene expression using qRT-PCR

**Figure 7:**
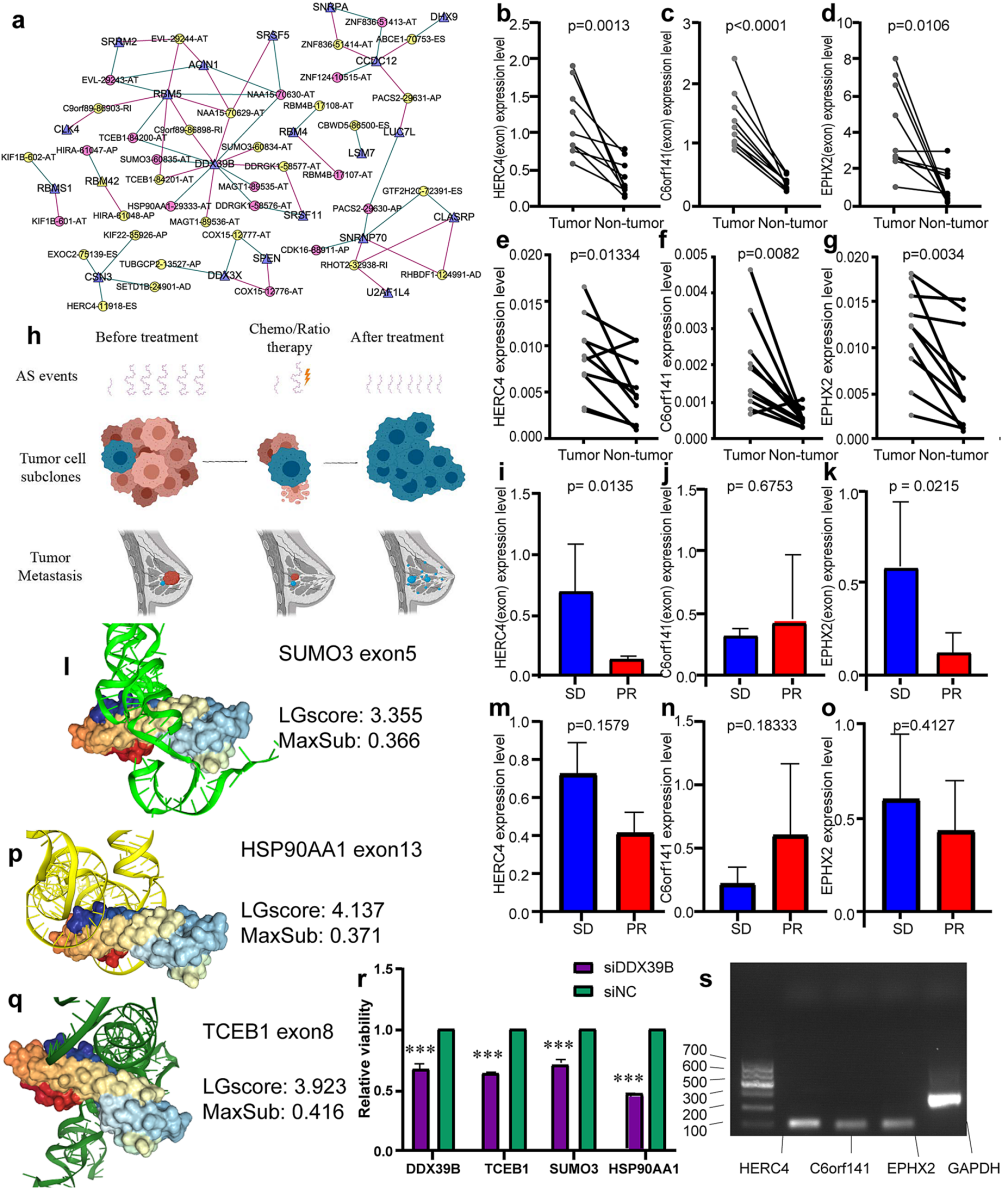
(**a**) The regulatory network of shear factors (SFs) and alternative splicing (AS) events. The purple triangle is SF, circle indicating AS events, in which yellow is a protective event and pink is a dangerous event; red lines represent AS events that are positively related to SF, and green lines represent AS events that are negatively related to SF. (**b**–**g**, **i**–**k**, **m**–**o**) Comparison of gene- and exon-expression level in breast cancer (BC) and adjacent breast tissue, expression level in the partial response (PR) and stable disease (SD) groups. (**b**–**d**) Exon-expression level of HERC4, C6orf141, and EPHX2 in BC and adjacent breast tissue; (**e**–**g**) gene-expression level of HERC4, C6orf141, and EPHX2 in BC and adjacent breast tissue; (**i**–**k**) exon-expression level of HERC4, C6orf141, and EPHX2 in SD and PR group; (**m**–**o**): gene-expression level of HERC4, C6orf141, and EPHX2 in SD and PR group. (**h**) Schematic diagram of the role AS events plays in the generation of the subclone in cancer. In AS events, the long purple wavy line represents AS events with relatively good prognosis, while the short wavy line represents AS events with poor prognosis. In tumour cell subclones and tumour metastasis, red cells and tissues showed a good prognosis, while blue cells and tissues showed a poor prognosis, suggesting that AS events with a poor prognosis played an important role in the recurrence of cancer after treatment. (**l**, **p**, **q**) Docking model for DDX39B and AS events. (**l**) DDX39B and SUMO3 exo5; (**p**) DDX39B and HSP90AA1 exo13; (**q**) DDX39B and TCEB1 exo8. (**r**) Expression of node genes and downstream genes after DDX knockdown. The purple represents siRNA specific against DDX39B group, the green represents control siRNA group, *** represents *P* < 0.001. (**s**) Agarose gel analysis of cDNA-based PCR product.

The exon-level and gene expression of C6orf141, HERC4, and EPHX2 was assessed by qRT-PCR and shown to be significantly higher in BC samples than in corresponding adjacent breast samples. We also investigated the relationship between exon-level and gene expression of C6orf141, HERC4, and EPHX2 and responses of patients after receiving chemotherapy was performed. Different significant exon-level expressions of HERC4 and EPHX2 were identified between the SD and PR group, but there was no significant difference in gene expression between this two groups. (Fig. 7b–g, i–k, and m–o).

### Network of SF-related AS events

According to Pearson’s correlation analyses, of 390 SFs, 21 genes were screened as their expressions were significantly associated with 39 prognosis-related AS events. SFs, such as DDX39B, CCDC12, RBM5, and SNRP70, were recognized as node genes that are crucial in the regulation network, in which DDX39B are negatively corrected with six protective AS events and positively corrected with six risky AS events (Fig. 7a).

### The function of DDX39B in IBC

In order to observe the potential role of DDX39B in breast cancer, we performed GSEA enrichment analysis. GO term enrichment analysis results showed that mRNA splice site selection, regulation of megakaryocyte differentiation, regulation of RNA splicing, regulatory RNA binding, and spliceosomal complex assembly were highly expressed in high-risk group, and ATP synthesis coupled electron transport, cellular respiration, establishment of protein localization to endoplasmic reticulum, large ribosomal subunit and structural constituent of ribosome were highly expressed in low-risk group (Fig. 5j). KEGG enrichment analysis results showed that spliceosome, Vegf signaling pathway, jak stat signaling pathway, non small cell lung cancer and notch signaling pathway were highly expressed in high-risk group, and amino sugar and nucleotide sugar metabolism, glycolysis gluconeogenesis, oxidative phosphorylation, pentose phosphate pathway and ribosome were highly expressed in low-risk group (Fig. 5k).

Molecular docking results showed that exons SUMO3(exon5), HSP90AA1(exon13) and TCEB1(exon8) bind most closely to DDX39B subunit 2FZT, as shown in Table 2. The docking results of these three exons and DDX39B subunit are shown in Fig. 7l, p, and q. Knockdown of DDX39B by siRNA and we found that the spliceosome expression of three downstream genes was down-regulated (Fig. 7r).

**Table 2:** Molecular docking parameters table.

AS events	LGscore	MaxSub
**HSP90AA1-29333-AT**	**4.137**	**0.371**
**TCEB1-84200-AT**	**3.923**	**0.416**
**SUMO3-60835-AT**	**3.355**	**0.366**
TCEB1-84201-AT	3.217	0.317
C9orf89-86898-RI	2.961	0.332
NAA15-70629-AT	2.843	0.345
NAA15-70630-AT	2.594	0.376
SUMO3-60834-AT	2.328	0.327
DDRGK1-58577-AT	2.314	0.352
MAGT1-89535-AT	2.287	0.381
DDRGK1-58576-AT	2.260	0.332
MAGT1-89536-AT	2.062	0.310

LGscore and MaxSub are computed as the quality parameters. LGscore lower than 1.5 and MaxSub lower than 0.1 indicate the quality of correct, LGscore between 3 to 5 and MaxSub between 0.5 to 0.8 indicate the quality of “good”.

The AS events that ranked list of top are shown in bold and selected for further research.

## Discussion

Recently, treatments for BC have been largely diversified and precise to individuals, which is based on the application of the gene profiles such as Oncotype DX^8^, MammaPrint^34^, and Endopredict^8^; however, these gene profiles are still in demand for development. First, the applicability of gene models is constrained in specific conditions; for example, the Oncotype DX is effective for patients with hormone-receptor in the early stage^8^. Moreover, they lack functions in the evaluation for the possibility of drug tolerance after chemotherapy, and there are limited studies on the detection of transcriptome variants.

It has been found that AS events play an important role in the tumorigenesis and development. Numerous studies have proved that AS and SFs are involved in the proliferation, invasion, and metastasis of cancer. For example, the aberrant expression of long isoform of Bcl-xl leads to cell growth and transformation in BC^35^. However, decreasing its expression by knocking down SRSF3 could trigger apoptosis and reverse anti-apoptotic action of the tumour cells, which is regarded as a promising therapy method^36^.

In fact, AS events are one of the leading causes of generation of the subclone in cancer. It is defined by groups of minor cell subpopulation that enhances the proliferation of cancer cells by competing with each other under environmental constraints and excluding the faster proliferating competitor subpopulations^37^ (Fig. 7h). Several AS variants have been used as identification markers for the subclone of BC and B-cell lymphoma^38^. The parent gene of SUV420H1 was reported to contribute to abrogate DNA repair and confer increased invasion and migration in neighbouring cells through chemokine signalling and modulation of integrins^39^. Thus, it is important to reveal the mechanisms of AS event and subclone heterogeneity in BC for effective treatments.

AS is also gaining much attention in therapy resistance. In chemotherapy, the BRCA2∆E5 + 7 and ∆40p53 isoforms perform acquisition of resistance to the DNA cross-linking drug mitomycin C^40^ and cisplatin^41^, respectively. In other areas, the CD19-∆2 variant leads to loss of CAR recognition site to prevent CART-cell targeting/killing of B-ALL cells. The BRCA1-∆11q splice variant contributes to PARPi resistance, and a small molecule inhibitor P1-B could silence its expression and regain the sensitivity to PARPi^42^.

In fact, several cancer prognostic evaluation models that are based on alternative splicing signatures have been established, including pancreatic ductal adenocarcinoma, kidney renal clear cell carcinoma, and glioblastoma, while none of these have been transformed for clinic application^13,23,24^.

In this study, by mining AS events data from TCGA database, we first obtained 2146 survival-related AS events among 1551 parent genes by univariate cox regression analysis. Subsequently, GSVA was performed on these parental genes, and it was found that the B cell receptor signaling pathway and the ERBB signaling pathway promote the development of IBC, and the Wnt signaling pathway and Gnrh signaling pathway inhibit the progression of IBC.

Gonadotropin-releasing hormone (GnRH) is metabolized by the proteolytic regulatory enzyme pyrrolidone carboxypeptidase (Pcp) and it regulates cell proliferation, apoptosis, and tissue remodelling and it has been proved to be one of the important hormonal factors in the pathogenesis of BC^43^. The ErbB receptor is a type of cell membrane receptor tyrosine kinase. In many forms of malignant tumours, especially tumours derived from the ectoderm (including prostate cancer and breast cancer), members of the ErbB family are overexpressed, amplified or mutated, they regulate cell proliferation, apoptosis and migration of tumour cells through Akt, MAPK, and other pathways^44^. B cell is recently proved to play an important role in the immune therapy of the cancer, the patient had a better effect on the treatment with immune checkpoint blockade (ICB) when B cells form a cell cluster called "tertiary lymphoid structures (TLS)" in the tumour, immune subgroup rich in B cells has the highest survival rate after receiving anti-PD1 monoclonal antibody treatment in the clinical trial phase^45^. Wnt signalling is involved in the initiation and progression of breast cancer by Wnt proteins combining with low-density-lipoprotein receptor-related proteins 5/6 (LRP5/6) and receptors Frizzled (FZD), and initiating β-catenin-dependent or -independent signalling pathways^46^.

We used LASSO and multivariate COX regression analysis to screen out a prognostic model containing 14 AS events. The AUCs value in the ROC curve was greater than 0.6, which indicates that the model we constructed has good stability. The results of independent prognostic analysis showed that the prognostic model we constructed can be used as an independent prognostic factor. Meanwhile, exons and gene levels of HERC4, EPHX2 and C6orf141 were confirmed to be highly expressed in IBC tissues, these three AS events were the first to be discovered in IBC, notably, the exon-level expression of HERC4 and EPHX2 is upregulated in the SD group, which corresponds with the findings that the skipping of these exons is a relative protective indexes. Therefore, it can be speculated that the high expression of exons corresponding to HerC4 and Ephx2 may be a risk factor for poor prognosis of breast cancer, and may be a more sensitive indicator than the parent gene.

HERC4 is known as a member of the ubiquitin proteasome system that promotes the ubiquitination of LATS1 to destabilize LATS1. High expression of HERC4 is associated with poor prognosis in patients with IBC^47^. The silence of EPHX2 could induce apoptosis in prostate cancer cells by reducing androgen receptor signalling^48^. C6orf141 could be silenced by ZNF217 overexpression, thereby promoting BC cell metastasis to bones^49,50^. Low C6orf141 expression was associated with poor prognosis in other cancers^51^. The exon-specific qRT-PCR is applied to detect AS events and has been proved necessary in evaluating gene expression at single exon level for transcriptional biomarker research, especially in detecting AS events, in which it performed equally well or even better compared to ordinary gene qRT-PCR^52^. We found that expression of HERC4(exon 23) and EPHX2(exon 7) was higher in SD group, this confirmed our results of bioinformatic analysis that exon skip of HERC4 and EPHX2 is are protective factors. This also proved that the expression of AS events changes dynamically in the progress of chemotherapy treatment and are various in individuals, which suggests that AS is a potential sensitive predictor and potential target of the treatment.

Recently, SFs that regulate the expression of AS have gradually proved to contribute to the development of IBC, and corresponding treatment strategies have gradually emerged. For example, synthetically modified oligonucleotides (SMOs) aim to block the recognition of specific slice site to prevent shifting to cancer-associated splice variants. It successfully helps cancer cells recover the sensitivity to ultraviolet B radiation and chemotherapy by altering the ratio of Bcl-xL to Bcl-xS^53^.

SF is crucial for the regulation of AS events. In this study, we have located DDX39B as a node gene for AS-SF network. DDX39B, an RNA helicase, is involved in various cellular processes. Its overexpression promotes the global translation and cell proliferation by upregulating pre-ribosomal RNA, which leads to oncogenesis^52^. DDX39B also regulates several AS events that are associated with the pathway of sphingolipid metabolism and poor prognosis of kidney renal clear cell carcinoma^26^. Interestingly, there are two AS events in our model, whose parent genes also play a role as SFs. RAB5B is an SF that inhibits BC stem cell-like cell migration and invasion^54^. HNRNPM is also found to regulate several AS events and indicate a poor prognosis with axillary lymph node metastasis^55,56^.

Knockdown of DDX39B by siRNA and we found that the spliceosome expression of TCEB1, SUMO3, and HSP90AA1 was down-regulated, which indicated that the interaction network we constructed had predictive accuracy. TCEB1 promote the invasiveness of prostate cancer cells by regulating expression of Ankyrinsare protein^57^. In IBC, NOTCH1 signalling activation depletes unconjugated SUMO3 and thus the sensitivity to perturbation of the SUMOylation cascade is increased which leads to the cell proliferation^58^. HSP90AA1, also known as eHsp90α, has been reported to promote tumor cell metastasis and tumor motility in different types of cancer, including IBC^59^. These literatures show that these three parental genes can promote tumor progression.

## Conclusion

In conclusion, we first established a prognostic model containing 14 AS events, and verified by experiments, found that HERC4 (exon 23) and EPHX2 (exon 7) can be used as potential therapeutic targets for TNBC, and then constructed the regulatory network of AS events and SFs, identified DDX39B as the key node of SF, siRNA verification found that DDX39B can be used as a potential target for breast cancer treatment, which provides new ideas for breast cancer treatment.

## Supplementary information

Supplementary Information.

## Data Availability

The datasets used and/or analysed during the current study are available from the corresponding author on reasonable request.

## Abbreviations

IBC: Invasive breast cancer
AS: Alternative splicing
SFs: Splicing factors
AA: Alternative acceptor site
AD: Alternative donor site
AP: Alternative promoter
AT: Alternative terminator
ES: Exon skip
RI: Retained intron
ME: Mutually exclusive exons
TCGA: The cancer genome atlas
qRT-PCR: Quantitative real-time reverse transcription polymerase chain reaction
siRNA: Small interfering RNA
PSI: Percent spliced in
GSVA: Gene set variation analysis
LASSO: Least absolute shrinkage and selection operator
ROC: Receiver operating characteristic
AUC: Area under the curve
GO: Gene ontology
KEGG: Kyoto encyclopedia of genes and genomes
PR: Partial response
SD: Stable disease
K–M: Kaplan–Meier
