# Supplementary material for: Transcriptome-wide analysis and modelling of prognostic alternative splicing signatures in invasive breast cancer: a prospective clinical study
Source: Sci Rep. 2020 Oct 5;10:16504. doi: 10.1038/s41598-020-73700-1 (PMC7536242; doi:10.1038/s41598-020-73700-1)
Supplement: Supplementary file 1 — Supplementary Information. [file 41598_2020_73700_MOESM1_ESM.docx]

**Supplementary information**

**Transcriptome-wide analysis and modelling of prognostic alternative splicing signatures in invasive breast cancer，a prospective clinical study.**

Linbang Wang^1,2+^, Yuanyuan Wang^1+^, Bao Su^2^, Ping Yu^1^, Junfeng He^1^, Lei Meng^1^, Qi Xiao^1^, Jinhui Sun^1^, Kai Zhou^2^, Yuzhou Xue^2^, Jinxiang Tan^1*^

^1^Department of Endocrine and Breast Surgery, The First Affliated Hospital of Chongqing Medical University, Chongqing 400016, China.

^2^Department of Orthopedic Surgery, The First Affiliated Hospital of Chongqing Medical University, Chongqing 400016, China.

^+^These authors contributed equally to this work.

^*^Corresponding Author: Jinxiang Tan, E-mail: tjx1202@163.com

Phone: 0086-023-89011496, Fax: 0086-023-89011496.

Additional File 1: Table S1. Multivariate prognostic model containing 93 survival- associated AS events

| id | coef | HR | pvalue |
| --- | --- | --- | --- |
| 15AA |  |  |  |
| SAFB\|46852\|AA | -4.563 | 0.010432 | 0.000374 |
| GPBP1\|72126\|AA | -15.013 | 3.02E-07 | 0.023821 |
| ENY2\|84887\|AA | 2.324 | 10.21854 | 0.024956 |
| LRP6\|20501\|AA | -18.714 | 7.45E-09 | 0.00033 |
| EEF1B2\|57144\|AA | -1.636 | 0.194817 | 0.017853 |
| ZFAND1\|84305\|AA | -17.072 | 3.85E-08 | 0.038966 |
| MFSD1\|67456\|AA | 13.459 | 700417.7 | 0.00412 |
| RPS21\|60075\|AA | 1.080 | 2.945133 | 0.05864 |
| CEP78\|86658\|AA | -2.655 | 0.070268 | 0.008561 |
| MYO19\|40482\|AA | -2.286 | 0.101639 | 0.061765 |
| SEC24D\|70448\|AA | 3.530 | 34.13233 | 0.006082 |
| CKLF\|36734\|AA | 5.936 | 378.4407 | 0.016365 |
| PLIN3\|46828\|AA | -50.151 | 1.66E-22 | 0.000679 |
| IST1\|37516\|AA | -19.641 | 2.95E-09 | 0.002518 |
| TTC23\|32613\|AA | -1.445 | 0.235698 | 0.003559 |
| 15AD |  |  |  |
| APEX1\|26450\|AD | 18.186 | 79077275 | 0.008371 |
| DCAF11\|26842\|AD | -4.031 | 0.017758 | 0.036418 |
| ATP6V0E2\|82210\|AD | -20.463 | 1.30E-09 | 0.002922 |
| TRIT1\|1922\|AD | 3.739 | 42.07518 | 0.003055 |
| TMEM161B\|72733\|AD | -1.653 | 0.191441 | 0.003127 |
| JMJD4\|10060\|AD | -5.501 | 0.004081 | 0.006593 |
| GEMIN7\|50399\|AD | -1.905 | 0.148895 | 0.000687 |
| ZFAND1\|84310\|AD | -2.360 | 0.094463 | 0.138171 |
| ZSWIM7\|39411\|AD | 2.885 | 17.89982 | 0.0598 |
| RAB3IP\|23354\|AD | 3.917 | 50.25613 | 0.000248 |
| BCL2L11\|54966\|AD | -2.225 | 0.108049 | 0.035107 |
| NOSIP\|50974\|AD | 5.155 | 173.2925 | 0.043491 |
| SPATA7\|28698\|AD | -8.774 | 0.000155 | 0.003337 |
| ABCG1\|60705\|AD | -3.845 | 0.021381 | 8.79E-05 |
| C7orf49\|81873\|AD | -2.846 | 0.058063 | 0.008982 |
| 12AP |  |  |  |
| RAB5B\|22326\|AP | -6.029 | 0.002408 | 0.014143 |
| RIPK4\|60674\|AP | -5.867 | 0.002831 | 0.009727 |
| LYST\|10361\|AP | -0.952 | 0.385796 | 0.030989 |
| RPS6KA1\|1282\|AP | 2.637 | 13.96919 | 0.00088 |
| LRCH4\|80959\|AP | -34.424 | 1.12E-15 | 0.000126 |
| CXorf40B\|90325\|AP | 5.636 | 280.2989 | 0.012292 |
| ADAT2\|77977\|AP | 0.897 | 2.451573 | 0.062183 |
| KPNA6\|1553\|AP | -3.874 | 0.020767 | 0.018773 |
| C10orf128\|11501\|AP | -7.331 | 0.000655 | 0.002385 |
| EXOC3\|71438\|AP | -1210.597 | 0 | 1.24E-05 |
| TJP3\|46727\|AP | 1.887 | 6.599164 | 0.014378 |
| COA1\|79329\|AP | -4.671 | 0.009359 | 0.058464 |
| 10AT |  |  |  |
| MASP2\|636\|AT | -2.855 | 0.057568 | 0.007061 |
| BMPR1B\|69987\|AT | -5.781 | 0.003085 | 3.16E-06 |

| ZNF586\|52337\|AT | 4.804 | 122.0036 | 0.004271 |
| --- | --- | --- | --- |
| C6orf141\|76449\|AT | 1.275 | 3.579446 | 0.003015 |
| IYD\|78140\|AT | -1.039 | 0.353836 | 0.018387 |
| USP15\|22828\|AT | -3.094 | 0.045329 | 0.006124 |
| ZNF616\|51409\|AT | -2.307 | 0.099513 | 0.051107 |
| NFS1\|59212\|AT | -1.815 | 0.162862 | 0.150249 |
| BCCIP\|13433\|AT | 4.219 | 67.98622 | 0.012419 |
| WWOX\|37672\|AT | -3.732 | 0.023938 | 0.000619 |
| 16ES |  |  |  |
| CSAD\|21962\|ES | -5.330 | 0.004842 | 2.48E-07 |
| HNRNPM\|94942\|ES | -7.465 | 0.000573 | 0.011914 |
| FBXO28\|9933\|ES | -17.708 | 2.04E-08 | 0.016134 |
| ITPR1\|63015\|ES | -56.597 | 2.63E-25 | 1.45E-06 |
| HERC4\|11918\|ES | -30.567 | 5.31E-14 | 2.48E-05 |
| EPHX2\|83166\|ES | -12.307 | 4.52E-06 | 0.013948 |
| SUV420H1\|17300\|ES | -7.826 | 0.000399 | 0.01385 |
| NKG7\|51323\|ES | -188.067 | 2.11E-82 | 0.000465 |
| LPXN\|16012\|ES | -5.496 | 0.004105 | 0.004685 |
| ATL2\|53253\|ES | -4.597 | 0.010082 | 0.061302 |
| SLC37A3\|81990\|ES | -13.966 | 8.60E-07 | 0.000554 |
| MAZ\|35942\|ES | 2.045 | 7.730314 | 0.069469 |
| BTN3A2\|75630\|ES | -2.687 | 0.068085 | 0.007789 |
| CD74\|74077\|ES | 6.957 | 1050.828 | 0.001624 |
| KIAA0430\|34169\|ES | -5.868 | 0.002828 | 0.000244 |
| REEP5\|72996\|ES | -1.597 | 0.202404 | 0.103409 |
| 10ME |  |  |  |
| CPSF7\|99751\|ME | -2.023 | 0.132318 | 0.001094 |
| CCDC53\|106010\|ME | -1.469 | 0.230144 | 0.019848 |
| UBE2J2\|116089\|ME | 1.796 | 6.025231 | 0.002057 |
| GOLT1B\|92984\|ME | 1.615 | 5.028475 | 0.004297 |
| PTK2\|98071\|ME | 3.239 | 25.52048 | 0.014363 |
| TBC1D5\|63665\|ME | -1.362 | 0.256147 | 0.05232 |
| SRGAP1\|93242\|ME | 2.207 | 9.088842 | 0.029949 |
| COPS5\|115459\|ME | 1.998 | 7.37426 | 0.000723 |
| RAB28\|265743\|ME | 3.096 | 22.11674 | 0.050809 |
| ANKRD10\|105827\|ME | 2.055 | 7.810599 | 0.051789 |
| 15RI |  |  |  |
| ZNF275\|90421\|RI | -10.072 | 4.23E-05 | 0.001825 |
| LAT\|35919\|RI | -51.664 | 3.65E-23 | 1.34E-05 |
| NPIPB5\|35570\|RI | -1.387 | 0.249773 | 0.040655 |
| PCDHB11\|73790\|RI | -4.559 | 0.010474 | 0.000783 |
| PRX\|49900\|RI | 0.787 | 2.195926 | 0.092546 |
| GLI4\|85407\|RI | -1.394 | 0.247959 | 0.023487 |
| AGPAT2\|88193\|RI | 14.826 | 2747249 | 0.013402 |
| GLYR1\|33862\|RI | -5.659 | 0.003484 | 0.006742 |
| COPZ1\|22159\|RI | -1.529 | 0.216794 | 0.042312 |
| NABP2\|22408\|RI | 1.834 | 6.257402 | 0.153003 |
| ZNF576\|50223\|RI | -1.414 | 0.243182 | 0.010366 |
| AK9\|77206\|RI | -2.911 | 0.054425 | 0.016315 |

| RRP8\|14157\|RI | -2.897 | 0.05517 | 0.052663 |
| --- | --- | --- | --- |
| RBMX\|90220\|RI | -1.506 | 0.221826 | 0.046417 |
| PHTF1\|4284\|RI | 1.558 | 4.750736 | 0.009369 |
